# Necesidades de cuidado paliativo del paciente con falla cardiaca: un estudio mixto*

**DOI:** 10.15649/cuidarte.2539

**Published:** 2023-05-28

**Authors:** Lucely Marisel Fiscal-Idrobo, Priscilla Ospina-Muñoz, Lina María Vargas-Escobar, Maria Cilia Rincon-Buenhombre

**Affiliations:** 1 . Universidad El Bosque, Bogotá, Colombia. Email: lfiscal@unbosque.edu.co Universidad El Bosque Universidad El Bosque Bogotá Colombia lfiscal@unbosque.edu.co; 2 . Universidad El Bosque, Bogotá, Colombia. Email: pospinam@unbosque.edu.co Universidad El Bosque Universidad El Bosque Bogotá Colombia pospinam@unbosque.edu.co; 3 . Universidad El Bosque, Bogotá, Colombia. Email: lmvargase@unbosque.edu.co Universidad El Bosque Universidad El Bosque Bogotá Colombia lmvargase@unbosque.edu.co; 4 . Universidad El Bosque, Bogotá, Colombia. Email: maria.rincon@shaio.org Universidad El Bosque Universidad El Bosque Bogotá Colombia maria.rincon@shaio.org

**Keywords:** Evaluación de Necesidades, Cuidados Paliativos, Insuficiencia Cardíaca, Cuidadores, Grupo de Atención al Paciente, Needs Assessment, Palliative Care, Heart Failure, Caregivers, Patient Care Team, Determinado de Necessidades de Cuidados de Saúde, Cuidados Paliativos, Insuficiencia Cardíaca, Cuidadores, Equipe de Assistencia ao Paciente

## Abstract

**Introducción:**

La presencia de signos y síntomas físicos, psicosociales y espirituales, deben ser identificados y controlados por medio de la atención en cuidados paliativos que proveen los equipos y profesionales de la salud.

**Objetivo:**

Identificar las necesidades de cuidado paliativo de las personas con falla cardíaca, sus cuidadores y el equipo multidisciplinario de una unidad de falla cardiaca.

**Materiales y Métodos:**

Estudio mixto, con diseño transformativo secuencial (DI-TRAS), que inicio con una fase cuantitativa en la que se aplicaron los instrumentos: Edmonton, Facit-Sp-12 y el índice de Barthel. La segunda fase cualitativa se realizó con 3 grupos focales en los que participaron 7 pacientes, 8 cuidadores y 12 profesionales del equipo multidisciplinario. El estudio tomo como guía orientadora la teoría del manejo de síntomas desagradables de Elizabeth Lenz.

**Resultados:**

Se identificaron necesidades de cuidado paliativo desde lo fisiológico: edema, fatiga y disnea; psicológico: actitud frente a la vida y disfrutar de pasatiempos y factores situacionales: como dependencia del cuidador y redes de apoyo, los cuales permitieron su comprensión a partir de la teoría de Lenz.

**Conclusiones:**

Las necesidades de cuidado paliativo en pacientes con falla cardiaca, se presentan desde un marco de síntomas que son percibidas por pacientes, cuidadores y el equipo de salud. Se requieren abordajes integrales que mejoren la experiencia del síntoma.

## Abstract (en)

**Introduction:**

The presence of physical, psychosocial, and spiritual signs and symptoms should be identified and managed through the palliative care that health care teams and professionals pro vide.

**Objective:**

To identify the palliative care needs of people with heart failure, their caregivers, and the multidisciplinary team of a heart failure unit.

**Materials and Methods:**

A mixed-method study with a sequential transformative design (DITRAS, for its acronym in Spanish) was conducted. It began with a quantitative phase in which the Edmonton Symptom Assessment Scale (ESAS), the Therapy-Spiritual Well-Being Scale (FACIT-Sp-12), and the Barthel Index were used. The qualitative phase was conducted with three focus groups involving seven patients, eight caregivers, and twelve health professionals from the multidisciplinary team. Elizabeth Lenz's Theory of Unpleasant Symptoms was used as a guideline for this study.

**Results:**

Physiological (edema, fatigue, and dyspnea) and psychological (attitude towards life and enjoyment of hobbies) palliative care needs and situational factors (caregiver dependence and support networks) were identified and could be understood through Lenz's theory.

**Conclusions:**

Palliative care needs in patients with heart failure are presented under a framework of symptoms that patients, caregivers, and the health care team perceive. Comprehensive approaches are required to improve symptom experience.

## Resumo

**Introdujo:**

A presenta de sinais e sintomas físicos, psicossociais e espirituais deve ser identifi cada e controlada por meio de cuidados paliativos prestados por equipes e profissionais de saúde.

**Objetivo:**

Identificar as necessidades de cuidados paliativos de pessoas com insuficiencia cardíaca, seus cuidadores e a equipe multiprofissional de uma unidade de insuficiencia cardíaca.

**Materiais e Métodos:**

Estudo misto, com desenho sequencial transformativo (DITRAS), que iniciou com uma fase quantitativa na qual foram aplicados os instrumentos: Edmonton, Facit-Sp-12 e o índice de Barthel. A segunda fase qualitativa foi realizada com 3 grupos focais nos quais participaram 7 pacientes, 8 cuidadores e 12 profissionais da equipe multidisciplinar. O estudo tomou como diretriz a teoria de controle de sintomas desagradáveis de Elizabeth Lenz.

**Resultados:**

As necessidades de cuidados paliativos foram identificadas do ponto de vista fisiológico: edema, fadiga e dispneia; psicológicos: atitude em relagáo a vida e gosto de hobbies e fatores situacionais: como dependencia do cuidador e redes de apoio, que permitiram sua compreensáo com base na teoria de Lenz.

**Conclusóes:**

As necessidades de cuidados paliativos em pacientes com insuficiencia cardíaca sáo apresentadas a partir de um quadro de sintomas que sáo percebidos pelos pacientes, cuidadores e equipe de saúde. Abordagens abrangentes sáo necessárias para melhorar a experiencia do sintoma.

## Introducción

La falla cardiaca (FC) es definida como un síndrome clínico que se presenta en un clúster de signos y síntomas típicos de la enfermedad, como disnea, fatiga, edemas de miembros inferiores, congestión pulmonar, entre otros; que sugieren el deterioro del gasto cardiaco y/o sobrecarga de volumen cardiaco^1^. La principal problemática de vivir con FC se refleja en las limitaciones funcionales, la polifarmacia y la disminución de la calidad de vida^2^; de donde surgen necesidades de cuidado paliativo debido a la alta morbilidad, hospitalizaciones frecuentes, dilemas en la toma de decisiones, expectativas de supervivencia y de la exigencia de la enfermedad a los pacientes, sus cuidadores y el sistema de salud^3^. Estas necesidades se resumen en la presencia de signos y síntomas físicos, psicosociales y espirituales, que son controlados por medio de la atención en cuidados paliativos que proveen los equipos de salud^4^ y en donde la integración temprana de estos cuidados, juega un papel importante en la calidad de vida de la persona. Así mismo, la participación de los cuidadores es fundamental ya que proporcionan apoyo al paciente influyendo en muchos casos en la adherencia al tratamiento, el cuidado y su calidad de vida^5^, por lo cual se hace necesario que dentro de los cuidados paliativos se integre también al cuidador.

El Atlas Global de Cuidado Paliativo expone que hay poblaciones de adultos mayores de 60 años, con enfermedades crónicas cardiovasculares, que tienen necesidades de cuidados paliativos al final de vida. Las necesidades de cuidado paliativo son definidas como un sentimiento ligado a la experiencia de un déficit o carencia que puede mostrar un alcance y complejidad variable^6^, donde se maneja un esfuerzo por suprimirlo a través de las intervenciones y cuidados de los pacientes en fin de vida o con enfermedades crónicas donde ya no es posible intervenir de manera curativa y donde el vivir lo mejor posible con calidad de vida es el objetivo, el paciente es visto no solo como persona sino también su entorno donde se mencionan factores influyentes como fisiológicos, psicológicos y situacionales no dejando de lado el rol físico, cognitivo y social de la persona enferma y su familia. Gran parte de los programas de FC en Colombia, no cuentan con un equipo integrado de cuidados paliativos que aborden de manera integral y temprana las necesidades paliativas del paciente y su familia dado que no son reconocidas^7^.

La FC condiciona al paciente a vivir con varios síntomas de manera simultánea, con la certeza que la descompensación de uno de ellos puede desencadenar un nuevo síntoma y mayores necesidades de cuidado paliativo. En este sentido, la teoría de los síntomas desagradables de Lenz proporciona una guía para el análisis multifactorial de la situación, al adoptar tres conceptos principales que son los síntomas, el resultado y el rendimiento de la experiencia del síntoma, estos se relacionan a su vez, con factores de riesgo fisiológico, psicológico y situacionales que predisponen la manifestación de los síntomas y la naturaleza de la experiencia de estos^8^. Esta perspectiva teórica, permite contemplar todo el clúster de síntomas, considerando la variabilidad de las percepciones y molestias que varían de una persona con FC a otra, según su condición individual y las circunstancias que enfrentan con la enfermedad.

Enfermería es una disciplina que participa en el proceso de cuidado paliativo e integra las acciones de todo el equipo de salud, con el fin de reducir el sufrimiento y a mejorar la calidad de la vida de los pacientes con FC y de sus familias, mediante una evaluación, identificación y gestión del dolor; sus necesidades físicas, sociales, psicológicas, espirituales y culturales; en este contexto el presente estudio tuvo como objetivo, identificar las necesidades de cuidado paliativo (presencia del síntoma, evaluación funcional y grado de dependencia del paciente) desde las perspectivas de los pacientes, cuidadores y equipo multidisciplinario de un programa de FC.

## Materiales y Métodos

Estudio mixto que siguiendo la metodología propuesta por Cresweell^9^ y Sampieri^10^, empleó procesos de recolección, análisis y vinculación de datos cuantitativos y cualitativos con un diseño transformativo secuencial (DITRAS) que involucró dos etapas de recolección de los datos. La fase inicial fue la cuantitativa, en la que se aplicaron 3 instrumentos para identificar las necesidades de cuidados paliativos de los pacientes con FC. La segunda fase cualitativa fue desarrollada con tres grupos focales, en las que se pudo conocer las percepciones de pacientes, familiares y equipo multidisciplinario sobre estas necesidades, la interpretación de la información de ambas fases se integró y se teorizó a la luz de la perspectiva teórica de los síntomas desagradables de Lenz^8^. La base de datos se encuentra disponible en el repositorio del Sevier^11^.

El estudio se realizó en una unidad de FC de una institución de Bogotá, especializada en cardiología. La fase cuantitativa se desarrolló desde un abordaje observacional descriptivo, entre el periodo comprendido entre febrero y marzo de 2019, en el que participaron 56 pacientes con FC en estadío II, III y IV, que fueron captados en un muestreo por conveniencia y a quienes se les administraron los siguientes instrumentos: el cuestionario de evaluación de síntomas de Edmonton (ESAS), que establece la presencia y severidad del síntoma. Este incluye 10 ítem con aspectos físicos y psicológicos. Se presentan en escala tipo likert de 1 a 10, donde 1 es ausencia del síntoma y 10 mayor severidad del síntoma^12^. Krikorian y Limonero utilizaron la escala en su estudio Factores asociados a la experiencia de sufrimiento en pacientes con cáncer avanzado realizado en Medellin^13^. La escala FACIT SP12 (versión 4) mide la evaluación funcional para el tratamiento de enfermedades crónicas; contempla 5 áreas: estado físico general de salud con 7 ítems, bienestar social con 7 ítems, bienestar emocional con 6 ítems, bienestar funcional con 7 ítems y bienestar espiritual: 12 ítems. Se presentan en escala tipo likert de 0 a 4, donde 0 es nada y 4 es muchísimo^14^.

Dado que estos dos instrumentos no cuentan con puntos de corte claramente establecidos en las versiones validadas para Colombia, se empleó el procedimiento de frecuencia de raíces acumulativas de Dalenius y Hodges a partir de los datos obtenidos^15^. Este método permitió establecer un estrato en la escala ESAS de bajo con puntajes entre 0 y 34 y altos entre 35 y 100; y para la escala FACIT SP12: estado físico general bajo puntajes de 0 a 10 y alto de 11 a 28, bienestar social puntajes bajo de 0 a 13 y alto de 14 a 28, bienestar emocional bajo de 0 a 9 y alto de 10 a 24, bienestar funcional bajo de 0 a 12 y alto de 13 a 28 y bienestar espiritual bajo de 0 a 36 y alto de 37 a 48 puntos. Finalmente, el índice de Barthel mide el funcionamiento diario de una persona en las actividades de la vida diaria. El rango de posibles valores está entre 0 y 100. Cuanto más cerca de 0 está la puntuación de un sujeto, más dependencia tiene; cuanto más cerca de 100 más independencia^16^.

Además de la administración de los anteriores instrumentos, se consideró indagar sobre: la información sociodemográfica, el estado nutricional, la clase funcional (según NYHA-Clasificación funcional de la insuficiencia cardíaca)^3^, las comorbilidades y hospitalizaciones en los últimos seis meses, las cuales se tomaron de la historia clínica, previa autorización de la institución y los participantes.

Los criterios de inclusión para esta fase fueron pacientes mayores de 18 años, que estuvieran vinculados al programa de FC y que no tuvieran dificultades de comunicación, o deterioro cognitivo. Posterior a la aplicación de estos instrumentos, los datos fueron registrados en la plataforma Red-Cap y se analizaron en el software Stata versión15, utilizando estadística descriptiva. Los resultados permitieron identificar las necesidades paliativas de los pacientes con FC, además se identificó el grupo de pacientes que participarían en el grupo focal.

Culminada la fase cuantitativa, se inició la fase cualitativa, en la cual se desarrollaron 3 grupos focales de interés en el que participaron por conveniencia 7 pacientes (que estuvieron en la fase cuantitativa), 8 cuidadores familiares que se encontraban en la unidad ese día y otros que llegaron a la convocatoria realizada previamente. Participaron los 12 profesionales que hacen parte del equipo de atención de la unidad, (entre médicos, enfermeras, psicóloga, nutricionista y trabajadora social, vinculados laboralmente a la institución de salud); en el mes de mayo de 2019 y que aceptaron participar en el estudio. En esta fase se buscó profundizar a través de un dialogo conjunto entre los diferentes actores, sobre sus percepciones frente a las necesidades de cuidado paliativo identificadas durante la falla cardiaca y la forma en que ellos piensan, podrían ayudar a solventarlas desde su propia experiencia. Esta discusión estuvo orientada por una serie de preguntas derivadas de las reflexiones de la fase cuantitativa. Las tres sesiones estuvieron orientadas por una de las investigadoras, otra cumplió el rol de observadora no participante, quien efectuó el registro de apreciaciones en cada sesión y los demás miembros del equipo apoyaron la logística de la sesión. Las sesiones en promedio duraron entre 30 y 45 minutos, se grabaron en video y fueron transcritas en word.

El análisis de la fase cualitativa de tipo descriptivo se realizó un análisis de contenido, con el fin de sistematizar la información de los grupos focales según lo establecido por Krippendorff citado por Abela^17^, se predeterminaron las categorías teniendo en cuenta la perspectiva teórica de los síntomas desagradables de Lenz desde los factores fisiológicos, psicológicos y situacionales, luego de leer la transcripción de lo obtenido en cada grupo focal, de forma manual se determinaron las subcategorías en cada una de las categorías correspondientes.

El rigor del estudio se estableció por los criterios establecidos por Creswell^9^, el estudio fue aprobado por el Comité de Ética de investigación humana de la Fundación Clínica Shaio, se consideró como un estudio sin riesgo de acuerdo con la Resolución 8430 de 1993, tuvo en cuenta los principios establecidos en la declaración de Helsinki y la Ley 911 de 2004, los participantes firmaron el consentimiento previo a la realización del estudio.

## Resultados

La edad promedio de los pacientes con FC fue de 73 años, son de género masculino (64.29%), de estratos socioeconómicos variados entre 1 y 4 (92.85%), son pensionados (85.71%) y tienen una escolaridad de primaria (37.50%) o secundaria (30.41%), la fracción de eyección es de 20.5, con progresión al deterioro nutricional, 3 ingresos mínimos en 1 año a hospitalización (67.86%) y comorbilidades como las que se observan en la Tabla 1. No se encontraron diferencias estadísticamente significativas entre las variables sociodemográficas y características clínicas de los pacientes con FC y el ESAS o el FACITSp.

**Tabla 1:** Perfil sociodemográfico y características clínicas de los pacientes con FC

Variables	% (n =56)
Edad (Años) Media ± D. Estándar	±10,5 (73)
Género N (%)	
Masculino	64,29% (36)
Femenino	35,71% (20)
Procedencia	
Urbana	98,21% (55)
Rural	1,79% (1)
Escolaridad	
Ninguna	5,45% (3)
Primaria	37,50% (21)
Secundaria	30,41% (17)
Tecnológico, pregrado, posgrado	26.64% (15)
Ocupación	
Hogar (pensionado)	85,71% (48)
Estudia	1,79% (1)
Trabaja	12,50% (7)
Estrato Socioeconómico	
1 - 2	23,21% (13)
3 - 4	69,64% (39)
5 - 6	7,14% (4)
Régimen de Seguridad Social	
Contributivo	82,14%% (46)
Especial	17,86% (10)
Clase Funcional NYHA	
Estadio II	23,21% (13)
Estadio III	62,5% (35)
Estadio IV	14,28% (8)
Tipo de Falla Cardiaca	
Isquémica	41,07% (23)
Valvular	17,85% (10)
Isquémica y valvular	17,85% (10)
Otras	23.23% (13)
FEVI Media ± D. Estándar	15 ± 33,5 (20,5)
Marcadores Nutricionales	
Severidad	16,67% (9)
Progresión	31,48% (17)
Impresión Clínica	51,85% (30)
Ingresos hospitalarios con síntomas de Falla Cardiaca	
Ninguno	5,36% (3)
< 3 Último año	67,86% (38)
> 3 Último año	26,79% (15)
Comorbilidades	
Enfermedad oncológica	5,35% (3)
Enfermedad pulmonar crónica	16,07% (9)
Diabetes Mellitus	42,85% (24)
Enfermedad renal crónica	44-64% (25)
Enfermedad neurológica crónica	12,5% (7)
Otros	29 % (51.78)
Variables	% (n =56)
Capacidad Funcional (Escala Barthel)	
Grave (20-35)	1,79% (1)
Moderado (40-55)	10,71% (6)
Leve (> 60)	75,00% (42)
Independiente (=100)	12,50% (7)

Por otro lado, el estudio tomó tres conceptos principales de la teoría de Lenz: los factores de riesgo fisiológicos, psicológicos y situacionales que predisponen, la manifestación y naturaleza de la experiencia del síntoma^8^, la FC condiciona al paciente a vivir con varios síntomas al mismo tiempo y con la certeza de que la descompensación de uno de ellos puede desencadenar otro, el estudio permitió contemplar el clúster de síntomas de los pacientes con FC, considerando la variabilidad de las percepciones y molestias de una persona a otra, su individualidad y las diferentes situaciones que enfrentan con la enfermedad.

### Necesidades paliativas derivadas de factores fisiológicos de la FC

**Figura 1:**
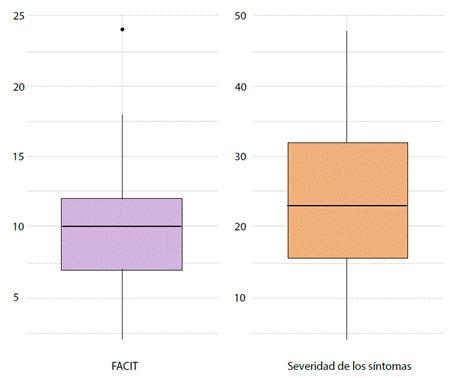
Pacientes con FC que presentaron alteraciones físicas de acuerdo a FACIT y ESAS

La edad, la fisiopatología de la enfermedad, las comorbilidades y el estado nutricional influyen en la experiencia de los síntomas, el cuidado paliativo va enfocado inicialmente en la intervención de esta dimensión física mediante tratamiento farmacológico y no farmacologico^1^. Las necesidades fisiológicas están representadas en una serie de síntomas que fueron identificados con la aplicación de la escala de ESAS, donde 25 pacientes (44.64%) presentaron alta severidad del síntoma. Igualmente, en el estado físico general de la escala FACITSp, donde 34 pacientes (60.71%) presentaron un bajo bienestar físico; la Figura 1 muestra la comparación entre ambas escalas. Los síntomas más frecuentes en los pacientes fueron: el dolor, la fatiga y la disnea. Otros síntomas como la falta de energía, las náuseas, la fatiga, la disnea, el insomnio y el edema también se hicieron presentes.

El dolor fue un síntoma expresado en el 54,8% de los pacientes desde 1 a 10 grados de intensidad Este síntoma puede influir en la trayectoria de la enfermedad y la agudización de otros síntomas, derivándose en una necesidad de cuidado paliativo diferente al clúster de síntomas característico de la FC.

Según la escala FACIT-Sp (ver Tabla 2), el 93% de los pacientes sienten falta de energía, (desde un poco a muchísimo 40%), lo cual indica la alta frecuencia de este síntoma en la FC, las náuseas son un síntoma que parece no relacionarse con la FC, pero que está presente en el 29% de los pacientes, otros síntomas reportados fueron la disnea y la fatiga que se presentan cuando van a dormir o realizan actividades cotidianas que demanden esfuerzo.

*“A veces tengo que dormir con cinco o hasta ocho almohadas, pero no deja de ser preocupante esa situación que en cualquier momento me puede dar, este dónde este” (Paciente 4).*

Por otro lado, la sensación de cansancio o de fatiga fue el síntoma más común en la escala ESAS en un 48,2% de los pacientes, con una intensidad de 8 a 10 grados (ver Tabla 1).

*“Yo quisiera hacer muchas cosas, pero no puedo, me fatigo afeitándome, viendo televisión no puedo hacer nada [..] cuando estoy acostado levanto mis brazos uno por uno, realmente yo no hago nada [...]" (Paciente 2).*

La fatiga para los pacientes se muestra con un cansancio extremo que le impide hacer actividades de autocuidado personal, limitándolo y haciéndolo cada vez más dependiente a medida que la deficiencia es más severa, siendo imposible la conciliación del sueño y afectando también su rol familiar.

*"[...] Tuve que salir para urgencias por que la fatiga fue tremenda, allá duré los siete días “(Paciente 4). “Yo le pongo animo a la vida, pero el corazón a veces se pone muy rebelde a no querer trabajar, ni uno duerme ni deja dormir" (Paciente 2).*

Las visitas hospitalarias son inevitables en los momentos cuando la fatiga es marcada en los pacientes, las estancias hospitalarias pueden ser largas.

Secundario a la fatiga, los pacientes sufrieron de insomnio. El 71.2% de los pacientes manifestaron un buen dormir desde un grado a 5 de la escala ESAS y solo el 4% refirió tener mucha dificultad para dormir; sin embargo, en la FACIT SP se reporta que al menos la mitad de los pacientes tiene problemas para dormir (42.8% poco o algo), (ver Tabla 2).

Para los cuidadores el síntoma más frecuentemente es el edema de acuerdo a los resultados del grupo focal. Para los cuidadores la retención de líquidos causada por la enfermedad es un síntoma que no olvidan, porque en casa deben medir la cantidad de líquidos que consumen los pacientes, la dieta hiposódica y la medicación que toman para controlarse.

*“Se le inflaman un poquito los pies y los ojos [...] Toca darle poquitica agua con lo que se toma y se come, además comer sin sal" (Cuidador 2).*

Los cuidadores consideraron un gran apoyo contar con el equipo de salud del hospital día en el manejo, evolución de los síntomas de los pacientes y en los casos que no es posible manejarlos en la casa, lo hacen desde hospitalización.

Otro aspecto valorado fue el relacionado en como la alteración del estado físico general (ver Tabla 2). La pérdida de la funcionalidad, los cambios en la calidad de vida con el avance de la enfermedad, las limitaciones, el deterioro y el manejo paliativo, son el interés del equipo de salud.

*“Nosotros generalizamos el programa de falla cardiaca, entonces hay un estadio A el paciente que está en riesgo de presentar falla cardiaca, [..] prevención de la enfermedad. Otro grupo estadio B tiene algo daño estructural pero aún no tiene síntomas, el abordaje se realiza es diagnosticado al paciente que ya tienen síntomas, [...], que se queda ahí en C o pasa al otro estadio D, que es candidato al abordaje paliativo [...]" (Cardiólogo).*

La necesidad de los profesionales por brindar un cuidado acorde a las necesidades de los pacientes ha hecho inevitable clasificar los pacientes para hacer el abordaje de promoción, prevención, manejo de síntomas y quirúrgico, en los casos que sea posible y paliativo en estadios avanzados de la enfermedad.

La ***falta de apetito*** resulto ser un síntoma que presentaron en alguna medida (82.1%) (ver Tabla 1). Los profesionales de la salud también comentaron problemas relacionados con la parte nutricional a medida que avanza la enfermedad:

*“Pues tú sabes que el paciente se deteriora más, pues ya no está comiendo. Te sientes limitada si hay necesidad de pasar una sonda, porque definitivamente el paciente no come nada, es muy complicado la enfermedad avanza" (Nutricionista)*

### Necesidades paliativas derivadas de factores psicológicos de la FC

Las necesidades de cuidado paliativo que desencadenan los factores psicológicos fueron: actitud frente a la vida y disfrutar de pasatiempos, espiritualidad, afrontamiento frente a la enfermedad, cambios en el estilo de vida y temor a la muerte. Según Lenz, el estado mental de la persona y la reacción afectiva a la enfermedad, son factores psicológicos que influyen en la experiencia de los síntomas. Considera algunas respuestas cognitivas como el grado de incertidumbre y el nivel de conocimiento que se tiene acerca de la enfermedad y los síntomas, las habilidades de afrontamiento y la disponibilidad de recursos que se tengan para lograrlo^6^. Para ello, psicología indaga e interviene en este nivel como parte del equipo multidisciplinario de cuidados paliativos, a través de la terapia cognitivo conductual y/o humanista^13^.

La Tabla 2 presenta la evaluación funcional para el tratamiento de enfermedades crónicas y bienestar espiritual (FACIT- Sp), donde se evidencia que las más bajas puntuaciones se obtienen en el estado físico general de salud y el bienestar emocional y la más alta en el bienestar espiritual.

**Tabla 2:** Evaluación funcional para el tratamiento de enfermedades crónicas y bienestar espiritualFACIT-Sp

Dimensiones	%	Bajo	%	Alto
Estado físico general de salud	60,71	34	39,29	22
Bienestar social y familiar	57,14	32	42,86	24
Bienestar emocional	58,93	33	41,07	23
Bienestar funcional	53,57	30	46,43	26
Bienestar espiritual	46,43	26	53,57	30
Total de la Escala	55,36	31	44,64	25

Las actitudes referidas por los pacientes se dan alrededor de los ***sentimientos hacia la vida y en cómo disfrutarla*** , que incluye los pasatiempos, estas actitudes son de tipo afectivo y emocional y son resultado de las relaciones con los cuidadores, su familia y con ellos mismos, se encontró que el 75% de los pacientes disfrutan nada, un poco o algo de la vida:

*“Hay momentos donde realmente no hago nada, no me llama la atención leer, ni que la mujer me hable, la deficiencia es tan severa [...] me gusta la música, me gusta estar conversando la visita etc. En la medida de la posibilidad eso no quiere decir que uno no quiera estar como cuando no teníamos absolutamente nada, vivir lo mejor” (Paciente 2).*

Con la presencia de la enfermedad, la restricción de actividades es común y la dependencia de los cuidadores para actividades diarias se aumenta, sentimientos de desasosiego por la pérdida de la funcionalidad, hacen que el ayer sea anhelado, momentos cuando aún no tenían la enfermedad. Los pacientes son dependientes de su familia quienes los ayudan en sus cuidados y en el sostenimiento financiero, son conscientes del nuevo rol familiar a desempeñar y el cuidar de sí mismos realizando los tratamientos.

*“Felizmente recién pensionada, con mucha esperanza porque llevo un año viniendo gracias a todo el equipo de salud y bueno estas reuniones y todo esto me llenan de vida.” (Paciente 3).*

Para los cuidadores es difícil que sus pacientes no hagan actividades de ocio que ocupen sus mentes y de la misma forma cuando se encuentran sensibles porque cualquier situación desencadena sentimientos de culpa y vulnerabilidad hacia sus pacientes.

*“Bueno mi esposo él siempre ha sido de mal genio, no voy a decir que toda la vida ha sido de mal genio. Y ahorita con su enfermedad, peor, sí, pero yo ya lo conozco y no le paro bolas” (Cuidador 7).*

Por otro lado, los profesionales de la salud brindan estrategias y planean intervenciones encaminadas no solo hacia los pacientes sino también involucrando a los cuidadores. Enfermería por ejemplo aborda el tiempo libre de los pacientes tratando de mantener su salud mental, minimizar el estrés y desarrollar otras habilidades y su creatividad.

*“Procuramos establecer estrategias orientadas hacia el paciente, haciendo los talleres, actividades, pintar, cantar, les llevamos música, todas esas estrategias apuntando al paciente esas siete horas que está ahí, se sienta bien y hacia el cuidador, tratando de abordar temas paliativos con objetivo también prepara ese cuidador para afrontar esas situaciones [...]” (Enfermera 1).*

Las limitaciones físicas por la enfermedad, la clase funcional NYHA estadio II (23.21%), estadio III y IV (76.8%), la edad promedio de la población fue de 73 años (ver Tabla 2), el hecho de que tengan que pasar tiempo acostados o sentados (66.1 % poco o algo) los puede hacer sentir enfermos en algún grado de intensidad, afectar la satisfacción que tienen frente a su calidad de vida (62.5% poco o nada) y del disfrute de pasatiempos (62.5% un poco o algo), como se muestra en la Tabla 2.

Por otro lado, a través de la espiritualidad se puede amortiguar la ***angustia de la muerte*** durante el progreso de la enfermedad, esta puede hacer ver la transición de la muerte más fácil, considerando que se ha cumplido una misión de vida y que se han dejado asuntos resueltos, el 67.8% de los pacientes encontraron consuelo dentro de sí mismos, y el 67.8% tienen un sentido de armonía interno.

De esta manera y de forma reciproca, tanto la enfermedad ha fortalecido su espiritualidad, como su espiritualidad les ha ayudado con la enfermedad, casi el total de los pacientes manifestaron que sienten que la enfermedad ha fortalecido su fe y creencias espirituales (90.9% mucho o muchísimo), encuentran fuerza (91.1% mucho o muchísimo) y consuelo en su fe o en sus creencias espirituales (91.1% mucho o muchísimo), ver Tabla 2, igual a lo percibido por lo cuidadores:

*“Por parte de mi esposo si, él ha cambiado mucho desde que él se ha visto tan grave, se ha acogido más a mi diosito, me dice a mi yo en cualquier momento me puedo ir y usted se queda va a quedar sola, mi dios es el único que sabe” (Cuidador 2).*

Para los profesionales de la salud la espiritualidad es una estrategia de afrontamiento hacia la enfermedad, donde se puede o no amortiguar la angustia en los pacientes o cuidadores hacia la situación de enfermedad.

*“Lo de la espiritualidad, es una necesidad, [...] como un refugio, como una forma para que seguramente se vea fortalecido, es más marcado esa relación con lo espiritual independiente de la religión [...] (Psiquiatra).*

En el proceso de ***afrontamiento de la falla cardiaca, es inevitable el cambio en el estilo de vida y los cambios en el estado de ánimo*** , la actitud y los roles que desempeñan en su núcleo familiar. Con la escala (ESAS) se evidencio que la tristeza y/o depresión es un síntoma frecuente (61,9%). El ***nerviosismo y/o la ansiedad*** se encuentran presentes en el 57,2% de los pacientes. El grado de satisfacción de enfrentar a la enfermedad (58.8% nada, algo o poco), mostrando cierta tendencia a la insatisfacción (ver Tabla 2), en preguntas como “estoy perdiendo las esperanzas en la lucha contra mi enfermedad”, se encontró en el 48.3%.

Para los cuidadores sobrellevar la enfermedad de los pacientes con FC implica estar expuestos a hospitalizaciones prolongadas, intervenciones quirúrgicas y manejos paliativos en casa porque la opción de trasplante ya no es posible y cambios al interior de la familia por la condición de enfermedad.

*"[...] Este final de año paso un problema con mis hijos porque a él lo mandaron a irse para un clima cálido, calientico porque la altura lo está perjudicando ya por el oxígeno, [..] entonces decidimos vender la casa, mis hijos pues se opusieron, él se puso mal porque ellos no querían y se pelearon con él [...]” (Cuidador 2).*

De otra manera, los profesionales de la salud sintieron que es necesario no asumir que el paciente y la familia sabe que solo se brinda un cuidado paliativo, el no explicarlo es un error. Al no comprenderse el propósito de cuidado paliativo no se permite satisfacer las necesidades de los pacientes tal como se mencionó a continuación:

*Las expectativas de las familias son supremamente altas[..] corroborando las expectativas ver sus historia clínica, uno dice, tiene toda la razón en tenerlas, ¿Doctor va a mejorar, de pronto ya no tenga que venir ..., en esa empatia que generamos con el paciente, no estamos concientizando de la expectativa real, [...] de pronto tampoco clara, enfática como ve es cierto, igual con los síntomas de depresión, de ansiedad, porque muchas veces ese tipo de síntomas se generan en esa falta de comprensión de que son manejo paliativo (Psicóloga)*

Padecer una enfermedad, supone un sentimiento constante de haber perdido el control de la vida y siempre se piensa en la muerte, se identificó que al 70.9% de los pacientes no les preocupa morir, en cambio 41% les preocupa que su enfermedad empeore.

*"Mi esposo, mi papá, mi hijo no sé qué, hay momentos en los que hay... la muerte es difícil es un punto muy denso y es muy doloroso uno hablar de eso. Eso es lo más fijo que uno tiene” (Cuidador 4).*

### Necesidades paliativas derivadas de factores situacionales de la FC

La teoría supone que los aspectos del ambiente social, como el estatus familiar, marital y del em pleo, el apoyo social, el acceso a la atención en salud, los recursos económicos y los estilos de vi-da, influyen en la forma en que la persona experimenta un síntoma y derivan también necesidades que son susceptibles de cuidado paliativo desde el trabajo social y terapia familiar8.

Se encontró que 85% de los pacientes tienen ***dependencia de leve a moderada y moderada*** (ver Tabla 1). Los pacientes continúan su proceso de enfermedad desde lo funcional, tratando de ser lo más autónomos posible, sin embargo, el requerimiento de un cuidador se hace necesario al final. Son los propios cuidadores quienes manifiestan su experiencia en el cuidado del paciente con FC:

*“Me toca hacerle de todo, afeitarlo bañarlo y vestirlo. no lo puedo dejar solo porque se cae" (Cuidador 9).*

Así mismo, manifiestan el impacto que tiene el cuidar a la persona con FC para su propia salud, ya que en muchos casos deben hacerse cargo de otras actividades y roles de desempeño. Uno de los elementos de este contexto es la perdida de la tranquilidad, la angustia y el estrés por parte del cuidador ante la adición de una nueva obligación y de enfrentarse a dificultades económicas.

*“Pues [.] yo ya tengo cansancio de cuidador, porque ya llevo mucho tiempo, así [..] no solo el problema de mi papá, sino también de la mama de mi esposo [...] me siento cansada, también con el tema del sueño pues ya tengo alteración de los nervios por eso" (Cuidador 4)*

*“Me ha afectado bastante, porque no he podido trabajar" (Cuidador 8).*

*“De verlo así, también yo me angustio y hasta me enfermo. Me estreso mucho" (Cuidador 7).*

Por otro lado, frente a los recursos para el cuidado de las personas con FC, se encontró que los ingresos hospitalarios por descompensación de síntomas fueron aproximadamente 3 en 1 año en un 67.86%; sin embargo, el 85.71% contaba con una pensión (ver Tabla 1). El factor económico influyo en la tranquilidad de las personas, así como el tener una buena entidad de salud; sin embargo, la gran mayoría se siente cortos para cubrir algunos gastos de transporte, copagos y medicamentos teniendo en cuenta que muchos de los pacientes lo hacen en transporte público.

*“Pues por mi parte mi esposo tiene su E.P.S y eso ayuda bastante, pero siempre se gasta plata viniendo a hospital día, se gasta siempre [.] que transporte [.]" (Cuidador 7)*

Los profesionales de salud identifican la solvencia económica y las redes de apoyo como la familia, como factores que ayudaron en el proceso y continuidad de los tratamientos de los pacientes con falla cardiaca tal como lo expresan a continuación:

*“Se trata de hacer el abordaje integral al paciente, sin embargo, a veces uno si se siente como corto, corto en el sentido de que el paciente, uno identifica unas necesidades grandes del paciente, por ejemplo, que no tengan de pronto la parte, la solvencia económica para poderle dar continuidad a su tratamiento" (Enfermera 1)*

*“Sí, es una persona que trabajaba y era el proveedor a nivel familiar entonces el piensa: ¿Que va a pasar con mi familia, con mis hijos?” (Cardiólogo)*

La familia se percibió como apoyo incondicional en el 73,2% de los pacientes, la mayoría no refieren lo mismo por sus amistades (46.43 % no refirieron apoyo) y 21.43 % muy poco (ver tabla 3). Los pacientes mencionan reiteradamente como su familia siempre los apoya:

*“La familia bien no me ha hecho falta nada[...] todo el mundo colabora tengo una lista para las personas que están dispuestas a acompañarme a cualquier sitio de la ciudad [...] hoy en día toda mi familia es un apoyo incondicional” (Paciente 2)*

Las barreras relacionado con el tratamiento mencionadas por parte de los cuidadores son los trámites administrativos para la autorización de procedimientos, lo que hace que deban realizar largas filas, con respecto a los medicamentos hacen la observación frente a la buena escritura de la formula:

*“Nosotros sí hemos bregamos un poquito porque con la EPS genera muchas trabas para los trámites administrativos sobre todo cuando hay procedimientos completos [..] con la EPS ha sido un poquito complicado, a veces los medicamentos los entregan en ciertas fechas, pero por ejemplo si queda algo que no esté bien escrito en la fórmula [..] no autorizan” (cuidador 4).*

## Discusión

Los pacientes con FC presentan necesidades paliativas fisiológicas representadas en síntomas percibidos por los pacientes, los cuidadores y el equipo de salud, para los pacientes los síntomas más frecuentes son disnea, fatiga e insomnio que se ven representados cuando van a dormir o realizan actividades cotidianas que demanden esfuerzo, secundario a la fatiga, tal como lo afirma Lenz, los síntomas pueden ocurrir solos o al mismo tiempo, lo más común es que se experimenten múltiples síntomas simultáneamente, lo que los hace difícil de diferenciar unos de los otros^18^, por otro lado, para los cuidadores el síntoma que más se menciona fue el edema, síntoma bastante percibido por ellos durante sus cuidados. Los estudios de Nordgren y Sorensen^19^, Lainscak y Keber ^20^, Schiff et al. ^21^, muestran que los pacientes tenían síntomas físicos y limitaciones que afectaban negativamente su calidad de vida, mencionaban que más del 80% presentan síntomas físicos como disnea, fatiga, edema, dificultades para dormir y dolor en el pecho, tal como los pacientes del presente estudio. Se reporta además en la literatura, que la complicación de los síntomas es el principal antecedente de reingreso hospitalario^22^^,^^23^^,^^24^.

Por otro lado, los profesionales de la salud mencionaron síntomas enfocados hacia la perdida de la funcionalidad y los cambios en la calidad de vida, el deterioro y el manejo paliativo, estudios de Ahmad et al., ^25^, Heo et al.,^26^ y Pisa et al.,^27^ encontraron que los pacientes, aunque todavía deseaban realizar actividades de la vida diaria como estaban acostumbrados, no podían hacer la rutina debido a los síntomas de la FC.

Los pacientes con FC presentan necesidades paliativas psicológicas representadas en actitud frente a la vida y disfrutar de pasatiempos, espiritualidad, afrontamiento frente a la enfermedad y cambios en el estilo de vida.

Con la presencia de síntomas propios de la enfermedad, la restricción de actividades es común y la dependencia de los cuidadores para actividades diarias se va generando^28^, sentimientos de preocupación por la pérdida de la funcionalidad, añoranza del ayer y alteraciones de desempeño de rol, es similar a lo encontrado por Ochoa^29^, Cortés^30^, Heo et al^26^., y Bernal et al^31^, dentro de las estrategias de afrontamiento la familia y el cuidador cobran importancia, se convierten en apoyo emocional y contribuyen en la búsqueda del sentido de vida.

Para los cuidadores es difícil cuando los pacientes se encuentran sensibles en diferentes situaciones de la enfermedad y de su propia cotidianidad porque cualquier situación desencadena sentimientos de culpa y vulnerabilidad relacionada con mala comunicación^32^^,^^33^, pérdida de la funcionalidad, aumento de peticiones emocionales de los pacientes^33^^,^^34^ y aumento de las comorbilidades^33^^,^^35^. Ochoa manifiesta que el proceso crónico de la enfermedad coronaria hace visible una actitud cansancio en el cuidador y que las relaciones familiares previas constituyen una ayuda en los momentos difíciles de la vida de los pacientes^29^.

Algo común entre los pacientes, cuidadores y los profesionales de la salud es cómo perciben la espiritualidad, una estrategia de afrontamiento, la fuerza espiritual que les permite a los pacientes y cuidadores tener esperanza. Según Ochoa “la continuidad de vida se convierte en un acto de fe y agradecimiento a Dios”^29^. Westlake et al.^36^, menciona que la espiritualidad les permite adaptarse ante una enfermedad crónica y las dificultadas presentadas.

Por otro lado, los pacientes describen la muerte como una certeza, tienen menos ansiedad o el temor a la muerte, porque han vivido la experiencia y son conscientes que en cualquier momento puede ocurrir. Esto es similar a lo encontrado por el estudio de Stromberg y Jaarsma^37^ donde la incertidumbre se daba en no saber cómo y cuándo acontecería la muerte. La muerte es vista como algo que se tenía que aceptar y aprender a afrontar. La adaptación a la enfermedad puede dar una explicación probable para la aparición de un cambio de respuesta, lo que significa que la muerte ya no da miedo.

Al referirse a las necesidades paliativas de tipo situacional de los pacientes la familia y las redes de apoyo social son importantes, esto es visto de forma similar por paciente, cuidadores y equipo de salud. Según Richardson la falta de apoyo social tiene un impacto negativo, muestran una mayor morbilidad y reingreso hospitalario38, además es un amortiguador para la carga del cuidador^33^^,^^39^ que les permite adaptarse más fácilmente su estilo de vida de acuerdo con la situación del paciente Family Partnership^33^^,^^40^.

Considerando el clúster de síntomas de este estudio, es importante reconocer que los profesionales de la salud, incluyendo a enfermería, deben mejorar la implementación de estrategias primarias, secundarias y terciaria para prevenir la aparición y progresión de la enfermedad, además de realizar una evaluación diagnóstica completa para determinar el tipo y la gravedad del síntoma, descubrir factores etiológicos corregibles, determinar el pronóstico, y guiar el tratamiento^41^. En este sentido, se hace indispensable la creación de equipos integrales de atención que manejen un lenguaje común porque no es suficiente conocer de los pacientes si al momento de integrar todas las intervenciones, el equipo se maneja por separado y no hay integralidad de la información, siendo esto clave de cuidado paliativo.

En este estudio el enfoque central es paciente con FC; sin embargo, los puntos de vista del paciente, cuidador y equipo de salud tuvieron diferencias o complementaros aspectos que los pacientes no consideraron. En los factores fisiológicos los pacientes refirieron mayor importancia a los síntomas que los llevaron a hospitalización o que le impedían realizar actividades básicas de su vida cotidiana y les genera disconfort; los familiares en cambio mencionaron los visualmente detectables y que generaban cambios en sus estilos de vida^42^^,^^43^, por su parte, los profesionales de la salud mencionaron los síntomas que generaban más dependencia y deterioro. En el aspecto psicológico los pacientes siempre mencionaron que a medida que la enfermedad avanzaba las actividades que podrían realizar eran cada vez menores, la familia y la espiritualidad fueron razón de apoyo y la muerte no era algo de temido sino en algunos casos esperado. Los cuidadores por el contrario refirieron que no era fácil asumir el rol teniendo en cuenta la frecuencia de las hospitalizaciones, el deterioro y dependencia y la afectación afecional^44^^,^^45^. El equipo de salud en cambio, siente la necesidad de tomar medidas que le permitan a los familiares y pacientes sobrellevar más fácil la enfermedad, ideando intervenciones que le brinden herramientas para que los pacientes mantengan la mente ocupada, tratando de beneficiar su salud mental, además de preparar a los familiares y pacientes para el manejo paliativo^46^.

Considerando los hallazgos en los síntomas de estos pacientes y la forma en que se presentan, se requieren de modelos de atención de Enfermería enfocados en esta población. Establecer un plan de acción para cuando los pacientes requieran admisión hospitalaria o manejo por atención domiciliaria en pro del confort y calidad de vida del paciente, ayudará tanto a pacientes, cuidadores y familiares a ajustar sus prioridades y estilos de vida.

El manejo de los pacientes con FC existe en otros contextos y está basado en modelos de atención especializados, que surgieron como estrategia para mejorar el pronóstico y calidad de vida de los pacientes^47^. Quizás la teleenfermería puede convertirse en una estrategia para tener acceso a pacientes en lugares de difícil acceso^48^. Esto teniendo en cuenta que el control sintomático de estos pacientes requiere del seguimiento por parte de Enfermería y del reconocimiento de su abordaje en estadíos avanzados, donde los cuidados de cuidados paliativos son apremiantes.

Es importante considerar que si bien gran parte de los síntomas, especialmente los físicos, son atendidos por los equipos de falla cardiaca, el abordaje de cuidados paliativos implica una atención integral que no busca solo manejar el síntoma físico sino todas las problemáticas asociadas de tipo emocional, social y espiritual, buscando una mejor calidad de vida y mejorar la experiencia del síntoma como lo plantea Lenz. El acompañamiento que se da no es circunstancial o momentáneo, sino que el seguimiento debe hacerse durante toda la trayectoria de la experiencia de la enfermedad hasta la muerte del paciente.

## Conclusiones

La FC a menudo está acompañada de otras patologías crónicas lo cual hace que presente múltiples síntomas asociados que deben ser abordados por los profesionales de la salud y los equipos de cuidado paliativo en las instituciones. El presente estudio permitió identificar síntomas como dolor, falta de energía, náuseas, fatiga, disnea, insomnio y edema desde lo fisiológico, sentimientos diversos hacia la vida, dependencia, apoyo familiar, sensibilidad, angustia ante la muerte o tristeza, entre otros. Desde lo psicosocial aspectos situacionales como la salud del cuidador, el aspecto económico, el apoyo desde el sistema de salud, entre otros que deben ser considerados desde la experiencia propia del paciente para ser abordados, como lo establece Lenz.

Por otro lado, desde la perspectiva de los profesionales de la salud del servicio de FC, se reconoce la importancia del cuidado paliativo y la falta de preparación para abordar muchas de las necesidades espirituales, emocionales, sociales, del duelo de pacientes y los cuidadores, aunque reconocen que desde lo fisiológico hacen un manejo adecuado e integrado en el que participan todos los miembros del equipo, es indispensable la integración de especialidades como algesiología, psicología y cuidados paliativos para abordar integralmente los pacientes con FC, es necesario también mejorar las habilidades de comunicación asertiva y afectiva con el paciente, para el abordaje adecuado de temas como el cuidado paliativo, pronostico, malas noticias y manejo del final de la vida.

La teoría de Lenz permite reconocer los síntomas fisiológicos, psicológicos y situacionales presen-tes en los pacientes con FC, lo cual permite a enfermería y a los equipos de cuidado paliativo establecer estrategias para mejorar la experiencia del síntoma y contrarrestar los efectos negativos que pueden producirse producto del deterioro de la enfermedad.
